# Persistent gene expression and DNA methylation alterations linked to carcinogenic effects of dichloroacetic acid

**DOI:** 10.3389/fonc.2024.1389634

**Published:** 2024-05-03

**Authors:** Gleta Carswell, John Chamberlin, Brian D. Bennett, Pierre R. Bushel, Brian N. Chorley

**Affiliations:** ^1^ Center for Computational Toxicology and Exposure, U.S. Environmental Protection Agency, Research Triangle Park, NC, United States; ^2^ Oak Ridge Institute for Science and Education, U.S. Environmental Protection Agency, Research Triangle Park, NC, United States; ^3^ Integrative Bioinformatics Support Group, National Institute of Environmental Health Sciences, National Institutes of Health, Department of Health and Human Services, Research Triangle Park, NC, United States; ^4^ Massive Genome Informatics Group, National Institute of Environmental Health Sciences, National Institutes of Health, Department of Health and Human Services, Research Triangle Park, NC, United States; ^5^ Biostatistics and Computational Biology Branch, National Institute of Environmental Health Sciences, National Institutes of Health, Department of Health and Human Services, Research Triangle Park, NC, United States

**Keywords:** transcriptomics (RNA sequencing), DNA methylation (5mC), dichloro acetic acid, mouse model, age, tumorigenesis, liver

## Abstract

**Background:**

Mechanistic understanding of transient exposures that lead to adverse health outcomes will enhance our ability to recognize biological signatures of disease. Here, we measured the transcriptomic and epigenomic alterations due to exposure to the metabolic reprogramming agent, dichloroacetic acid (DCA). Previously, we showed that exposure to DCA increased liver tumor incidence in B6C3F1 mice after continuous or early life exposures significantly over background level.

**Methods:**

Using archived formalin-fixed liver samples, we utilized modern methodologies to measure gene expression and DNA methylation levels to link to previously generated phenotypic measures. Gene expression was measured by targeted RNA sequencing (TempO-seq 1500+ toxicity panel: 2754 total genes) in liver samples collected from 10-, 32-, 57-, and 78-week old mice exposed to deionized water (controls), 3.5 g/L DCA continuously in drinking water (“Direct” group), or DCA for 10-, 32-, or 57-weeks followed by deionized water until sample collection (“Stop” groups). Genome-scaled alterations in DNA methylation were measured by Reduced Representation Bisulfite Sequencing (RRBS) in 78-week liver samples for control, Direct, 10-week Stop DCA exposed mice.

**Results:**

Transcriptomic changes were most robust with concurrent or adjacent timepoints after exposure was withdrawn. We observed a similar pattern with DNA methylation alterations where we noted attenuated differentially methylated regions (DMRs) in the 10-week Stop DCA exposure groups compared to the Direct group at 78-weeks. Gene pathway analysis indicated cellular effects linked to increased oxidative metabolism, a primary mechanism of action for DCA, closer to exposure windows especially early in life. Conversely, many gene signatures and pathways reversed patterns later in life and reflected more pro-tumorigenic patterns for both current and prior DCA exposures. DNA methylation patterns correlated to early gene pathway perturbations, such as cellular signaling, regulation and metabolism, suggesting persistence in the epigenome and possible regulatory effects.

**Conclusion:**

Liver metabolic reprogramming effects of DCA interacted with normal age mechanisms, increasing tumor burden with both continuous and prior DCA exposure in the male B6C3F1 rodent model.

## Introduction

The current default approach taken by the US Environmental Protection Agency (US EPA) when considering life-stage exposure is that cumulative exposure averaged over a lifetime is to be considered health protective when assessing exposure risk (1). However, high exposures may occur over a short-term window. In these cases, averaging these exposures over a lifetime may be inappropriate. It is therefore important to understand the linkage of life-stage stressors and latent adverse health outcomes. There are many examples of non-genotoxic exposures that increase susceptibility to cancer later in life (2–6). The mechanistic basis for this persistence can be multi-factorial, however the role of epigenetic dysregulation has increasingly been recognized as a common factor linked to cell proliferation, survival and invasion, immune response, genome instability, and energetics (7, 8) and has been proposed as an important hallmark of cancer. Therefore, a better mechanistic understanding of the epigenetic contribution to cancer biogenesis is a needed.

Dichloroacetic acid (*syn.* dichloroacetate, DCA) provides an interesting case study of latent carcinogenic effects from early-life exposure in mice. DCA is a halogenated acetic acid with low volatility that has previously been assessed by the US EPA because of its stable presence in drinking water as a byproduct of disinfection (9) and potential carcinogenic metabolite of the ground water contaminant, trichloroethylene (10). In male and female mice, as well as male rats, continuous exposure to DCA significantly increases the incidence of liver adenomas and carcinomas (9, 11–14), which led to the US EPA classifying it as a likely carcinogen. However, DCA is also recognized as a metabolic reprogramming agent and has been explored as therapeutic for diabetes, lactic acidosis, lipid/lipoprotein disorders, pulmonary arterial hypertension, and interestingly, for cancer (15). Given that typical amount of exposure in drinking water is much lower than calculated risk levels in humans (13, 16), it is unlikely these doses of concern are achieved due to environmental exposure. Therapeutic doses can reach much higher levels [25 mg/kg-day; (17)] and long-term deleterious effects could be of more legitimate concern, in addition to other recognized adverse effects such as peripheral neuropathy (18).

DCA-mediated mode-of-action for rodent liver cancer outcome is not entirely clear. The structure of DCA resembles that of pyruvate, which can bind and inhibit the activity of pyruvate dehydrogenase kinase [PDK; (19)]. PDK phosphorylates and subsequently inhibits the enzymatic action of the pyruvate dehydrogenase (PDH) complex, which is responsible for the conversion of glucose-derived pyruvate to acetyl-CoA. Activation of PDK reduces pyruvate shuttling to mitochondria (and subsequent acetyl-CoA-mediated activation of the TCA cycle) and instead favors pyruvate conversion to lactate. With DCA exposure, PDK is inhibited thereby activating the PDH complex and shunting glucose to the mitochondria and oxidative metabolism (20). This chemically induced bioenergetic shift can persist after exposure, therefore DCA is considered a metabolic reprogramming agent. However, it was also thought that this forced shift to mitochondrial oxidative metabolism may also be the cause of long-term oxidative burden, leading to cell injury, secondary DNA damage, and eventually cancer (21).

Importantly, our previous research confirmed that not only persistent DCA exposure but also short-term exposure early in life resulted in increased liver tumor burden (13, 22). These persistent tumorigenic effects occurred regardless of length of exposure length tested (ranged 4 wks to 93 wks). Most measured hepatic effects were transient, including mild hepatocellular necrosis, inflammation, and pigmentation, which resolved by 5 weeks after DCA exposure was removed (23). More persistent effects such as hepatocellular hypertrophy and cytoplasmic vacuolization (evidence of increased glycogenic storage) resolved by 26 weeks after DCA cessation. Overall, there was not strong evidence for classical genotoxic or non-genotoxic modes of action for latent carcinogenicity, leading to the hypothesis that persistent epigenetic alterations may be contributing to these latent effects. In this study, we utilize targeted RNA-sequencing and genomic-scaled DNA methylation methods using archived samples from this study to better understand the mechanistic basis for persistent effects of a brief carcinogenic exposure early in life.

## Methods

**Figure 1:**
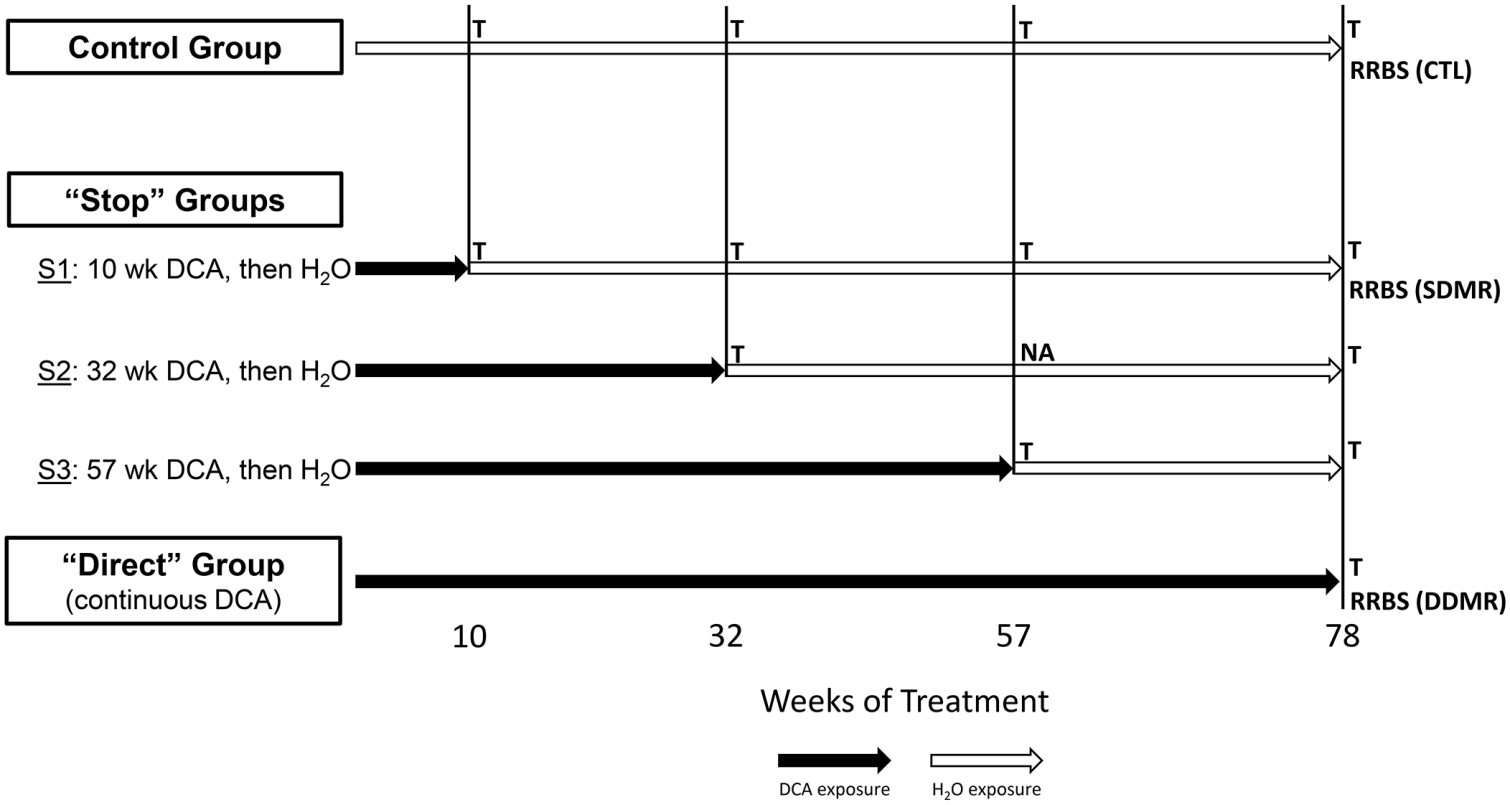
Study groups and sample overview. The stop-treatment mouse study design consisted of samples from four time courses of DCA exposure. The control group was given drinking water *ad libitum*, whereas mice in the “Stop” groups “S1”, “S2”, and “S3” had 3.5g/L DCA added for 10, 32, and 57 weeks, respectively. The “Direct” group was given DCA in drinking water for the length of the study (78 weeks). Liver gene expression was measured at each of these time points with the exception of “S2_57” weeks, where archived samples were not available (NA). The 1500+ TempO-seq assay was used to measure gene expression in FFPE samples indicated as “T”. Differentially Methylated Regions (DMRs) were determined by Reduced Representation Bisulfite Sequencing (RRBS) at the 78-week time point for the control (CTL), “S1” Stop group (SDMR) and the Direct group (DDMR) using frozen liver tissue.

The study utilized archival frozen and formalin-fixed paraffin-embedded (FFPE) samples that were ≥ 30 years old. Due to use in prior studies, we lacked frozen samples for all time points with the exception of 10 week Stop and Direct groups at 78 weeks (**Figure 1**), which were utilized for Reduced Representation Bisulfite Sequencing (RRBS, *see Methods below*). Gene expression was measured from FFPE samples using a target-based RNA-sequencing method that had been previously utilized to replicate findings in FFPE samples compared to matched frozen samples in a sister study of similar archival age (24). Because of the limitations of these lower quality samples, we used stringent criteria to identify potentially erroneous findings and, as a result, some groups had reduced sample numbers due to these quality control restrictions.

### Study design

As previously published, male B6C3F1 mice were treated with 3.5 g/L DCA in deionized water in a stop exposure study design over a period of 78 weeks (23). Mice were either provided only deionized water (control), continuous DCA (direct) or in a series of stop-dosage timepoints after which the mice were switched to deionized water for the duration of the experiment. The Stop group treatments were as follows: 10 weeks of DCA followed by 68 weeks of water (S1) which equates to an exposure period of early childhood thru adolescence, 32 weeks of DCA and 46 weeks of water (S2) which equates to an exposure period from early childhood to a mature adult and 57 weeks of DCA with 21 weeks of water (S3) which equates to an exposure period from early childhood to late middle age. The Direct group were exposed to 3.5 g/L for the entirety of the 78-week study (senior mice). At each timepoint (10, 32, 57 or 78 weeks), a subset of the control, Direct and Stop treatment block mice were humanely euthanized with CO_2_ following EPA approved Animal Care and Use Committee protocols. Portions of the livers were fresh frozen at each sacrifice timepoint along with the preparation of FFPE paraffin blocks. **Figure 1** outlines study collection points and treatments. FFPE-derived samples were used to measure gene expression and frozen samples were used for DNA methylation determination.

### Animal care

The sourcing, housing, and maintenance of the mice in this archived study have been extensively detailed in prior publications, along with dosing details and animal behavior notes (13, 22, 23). All procedures involving animal care were approved by an EPA Institutional Animal Care and Use Committee. Briefly, mice were received at age 21 days to the EPA AAALAC International accredited facility from Charles River Laboratories in Morrisville, NC, and acclimated for 7 days prior to treatment initiation. The animals were housed in polycarbonate cages in a laminar flow room and maintained under standard conditions (20 ± 2 C°; 40–60% relative humidity; 12 h light/dark cycle) and fed *ad libitum* Purina 5001 laboratory rodent diet (Purina Test Diets, St. Louis, MO). The study animals were monitored daily for health impacts. Water intake and body weight were recorded daily.

### Drinking water dosage

The target dose of 3.5 g/L (429 mg/kg-day) DCA (CAS 79-43-6, Aldrich, Milwaukee, WI) was based on the earlier carcinogenicity results observed in male mice (13, 22), and is equivalent to 65 mg/kg-day in humans (23). Although this dose was up to approximately 10^5^ times greater than DCA concentrations measured in finished drinking water samples (33–160 μg/l) (9) and approximately 2.6 times greater than doses used clinically in studies of children and adolescents with congenital metabolic diseases (12.5 mg/kg every 12 h) (19), this dose captured greater than 85% of the tumorigenic effect observed with lifetime exposure at the same 3.5 g/L dose (22). The actual dose was estimated based on intake, body weight and water DCA concentrations, and the daily water concentration (ml/kg-day) by cage was measured every 4-5 weeks beginning with week 4. Drinking water stocks were prepared every 12-14 days and checked by ultraviolet absorption at 231nm to confirm DCA concentration (Beckman DU Series 600 spectrophotometer, Beckman Coulter, Brea, CA). The stock water pH was adjusted to 6.8-7.2 with NaOH, stored at 4-6°C and changed every 5-7 days, based on studies showing DCA to be stable over 1-2 weeks (11, 14). As previously detailed, water consumption was initially 5-22% lower in the DCA drinking water groups, although by 52 weeks, water consumption was equivalent in all groups. The removal of DCA quickly returned water consumption to control levels, regardless of exposure length.

### DNA isolation

Approximately 25 mg of frozen liver tissue was cut on dry ice and immediately placed into a 2 ml vial with ceramic homogenization beads (MP Biomedicals, Lysing Matrix D) containing 360 µl of Qiagen buffer ATL (Qiagen GmbH, Hilden, Germany). The tissue was allowed to equilibrate to room temperature, 20 µl of 40 mg/ml Proteinase K was added and homogenized with a Precellys 24 homogenizer (Bertin Technologies, Villeurbanne, France) at 5500 rpm for 20 s. The homogenized sample tubes were placed in a 56°C thermomixer at gentle agitation for 3 h. After proteinase treatment, the samples were treated with RNAse A, and DNA was isolated using Qiagen’s DNeasy^®^ kit following the manufacturer’s instruction. The isolated DNA was checked for initial purity (A260/280 of ≥ 1.8, A260/230 ≥ 1.0) using a NanoDrop ND-1000 spectrophotometer (NanoDrop Technologies, Wilmington, Delaware). Yield was determined by Qubit broad range dsDNA assay kit and protocol (Life Technologies, Carlsbad, CA).

### Reduced representation bisulfite sequencing

DNA samples isolated from the frozen liver tissue (*n*=4-6) were analyzed for methylation on a genome-scale using a modified Reduced Representation Bisulfite Sequencing (RRBS) method (25, 26). Briefly, 500 ng DNA was digested overnight with MspI (300 ng) or TaqαI (200 ng). Each sample underwent an additional 2 hr digestion before restriction enzyme deactivation. Each restriction enzyme digested DNA sample was pooled and then bead purified using Agencourt Ampure XP beads (Beckman Coulter Life Sciences, Brea, CA), and Qubit quantitation was used to determine the amount of lambda (λ) for bisulfite methylation efficiency spike-in. End-repair and adapter ligation were performed with the KAPA Hyper Prep kit (Roche, Basle, Switzerland), along with a custom synthesized duplex adapter (Integrated DNA Technologies (IDT), Morrisville, NC). After overnight ligation and subsequent bead cleanup, the samples went through two rounds of bisulfite conversion and clean-up (Qiagen Epitect Bisulfite conversion kit, Germantown, MD). Library amplification was carried out using KAPA HIFI HotStart Uracil+ and IDT synthesized index and universal primer for 12 cycles, followed by bead cleanup. Library quality and yield were determined with the Agilent Bioanalyzer HS DNA kit (Santa Clara, CA), with the library size range predominately between 300 and 600bp. The libraries were sequenced on an Illumina NextSeq (San Diego, CA) by the National Health and Environmental Effect Research Laboratory (NHEERL) Genomics Research Core (GRC) at the US EPA using the NextSeq 500/500 75 cycle kit. Base calls were based on a Q-score of 30 for greater than 85% and a pass filter of ≥25M reads per sample. RRBS FASTQ files are available from NCBI GEO accession #GSE242665.

Differential methylation analysis was carried out using Bismark alignment (27). Briefly, sequencing adapters and read pairs with a mean Phred quality score of <20 were removed from reads using Trim Galore! (v 0.2.8). Bismark (v 0.14.3) aligned the reads to mm10 mouse genome assembly. Methylated and unmethylated cytosine were extracted from each CpG site and methylation percentages were derived, excluding any loci that were <10 reads and/or contained a known single nucleotide polymorphism. Average methylation percentages were calculated for the control, Direct and S1 78-week group samples by dividing the count of methylated reads by the total number of reads at each covered CpG site. Retained averaged methylation percentages contained >10 reads. All samples exhibited good bisulfite conversion rates (> 99%), alignment (>60% mapped to unique reference), and base quality scores (>96%, average = 98%). Differentially methylated regions (DMRs) were based on clustering of differentially methylated CpGs compared to control samples, which was used to generate DMRs based on the weighted methylation percentages from all CpGs in each region, as previously described (26). The final DMR list contained at least 3 CpGs within each DMR and an independent two-group Student’s *t*-test (*p*<0.05).

DMRs were mapped to regulatory features and transcription factor binding sites sourced from the Mouse Ensembl Regulatory Build, filtering for “liver:a8w” (release 90). CpG features were sourced from the UCSC annotation track database (ftp://hgdownload.cse.ucsc.edu/goldenPath/mm10/database/). Ensembl, Entrez and MGI identifiers were downloaded from BioMart data mining tool (https://useast.ensembl.org/info/data/biomart/index.html) to allow interconversion of identifiers. All DMRs for the Direct and Stop groups were linked to a single gene ID list based on nearest transcription start site using the R/Bioconductor package, GenomicRanges (28). Riken, GM- and pseudo genes were omitted from any gene or gene pathway subsequent analyses.

### FFPE derived mRNA sequencing

Archival samples were processed at BioSpyder, Inc. (Carlsbad, CA) using targeted Templated Oligo-Sequencing (TempO-Seq Mouse S1500+ Surrogate Assay, Mouse Tox v1.1, 3044 probes, 2755 genes) a liver-centric, sentinel gene, probe-based panel designed for high throughput predictions in toxicology (29). TempO-Seq performs well with older RNA-fragmented samples as compared to traditional RNA-Seq (24). Measurements were taken from direct lysates of archived mouse liver sections derived from FFPE blocks. Two to three sections of 15 µm thickness from each sample block were closely trimmed to remove excess paraffin and placed into -20°C storage until shipment overnight on dry ice to BioSpyder for library generation. Lysate purification, library preparation and sequencing were performed at BioSpyder using an established protocol (30). After passing quality control checks for total mapped reads in positive control samples (>1.5M mapped reads, we observed 4.6M reads), signal-to-noise ratio of the number of mapped reads in positive controls versus negative controls (>20:1, we observed 2077:1), and percentage of mapped reads in positive controls (>70%, we observed 95.9%), sequence data were aligned and matched to the probed gene for the assay. Gene count data were provided to EPA for further analysis. TempO-Seq FASTQ files and count data are available from NCBI GEO accession #GSE242665.Additional bioinformatic filtering was carried out to ensure quality of the data, necessitated by the age of the FFPE blocks (20+ years) and degradation of the resultant RNA. First, excessive deviation of gene expression ranking in individual samples from the average was assessed by Spearman rank regression. Any sample that exhibited an R^2^ value of less than 0.7 was excluded from further analysis (10 of 78 samples). A minimum *n* of 3 was required to keep a sample timepoint, although the majority of timepoints ranged from 4 to 6. Second, after median ratio normalization, genes with very low expression were eliminated from further analysis (those <3.4 (20%) geometric mean counts across all samples) as the final filter. Using these filtered samples and normalized genes, we determined differentially expressed genes from age-matched controls using DESeq2 (significance based on FDR ≤0.05) in Partek Flow software environment (Build 10.0.22.0524). A minimum fold change of greater than +/- 1.5 was set as an additional cut-off. Uniform Manifold Approximation and Projection (UMAP) was utilized as a general non-linear dimension reduction to visualize the first 15 components of Principal Components Analysis (PCA) for filtered, normalized count data before DEG determination, thereby capturing 99% of the variation of the filtered S1500+ gene expression data.

Further gene pathway analyses sets were carried out using Ingenuity Pathways Analysis (IPA) (QIAGEN Inc., https://www.qiagenbioinformatics.com/products/ingenuitypathway-analysis). Some visualizations were generated using R packages Plotly for bubble chart generation (v.4.10.1 https://github.com/plotly/plotly.R), VennDetail for Venn and UpSet plot generation (v 4.3 https://github.com/guokai8/VennDetail), and shinyCircos for Circos plot generation (v 3.3.3, https://github.com/venyao/shinyCircos).

## Results

### Gene expression overview

**Figure 2:**
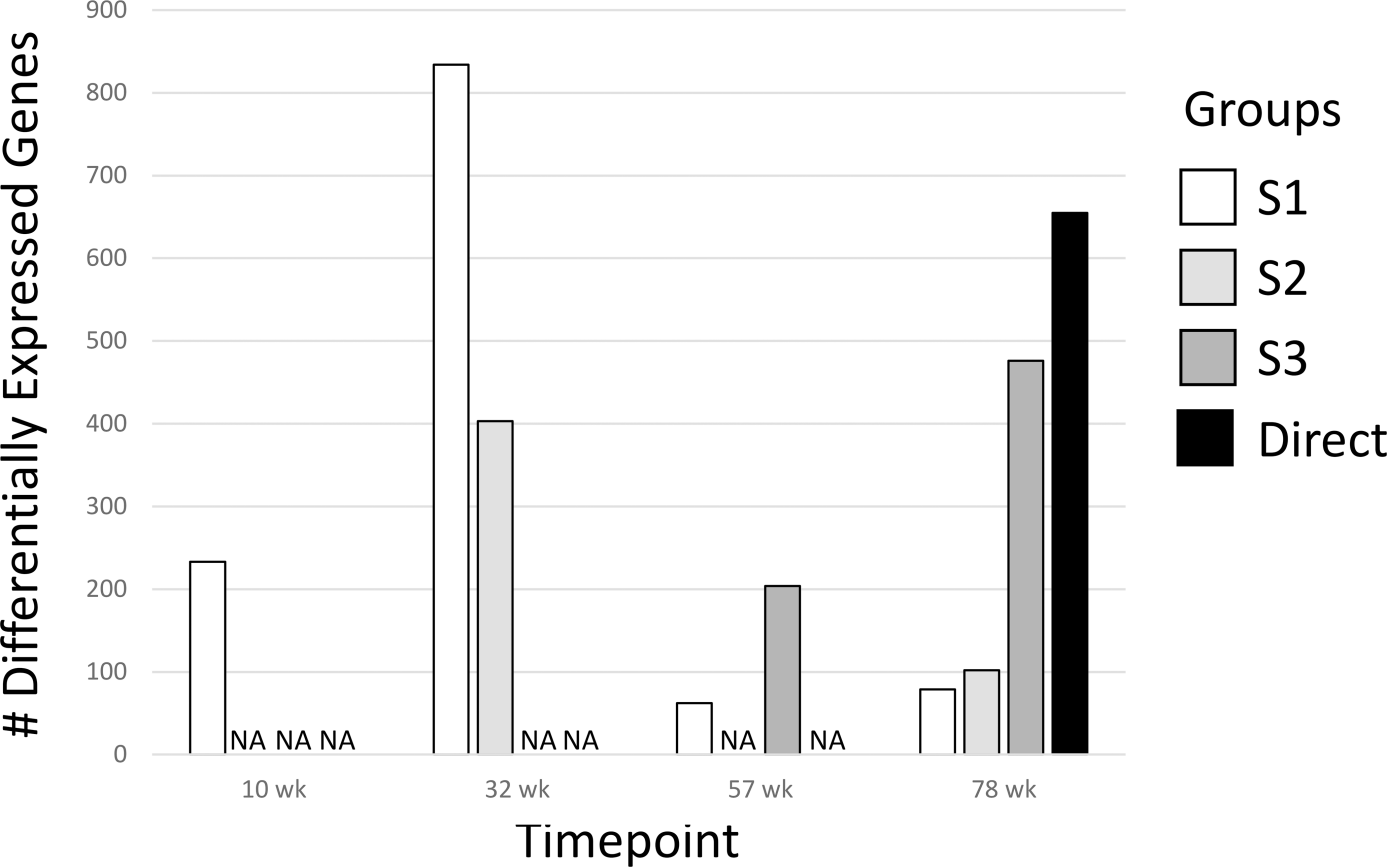
Differentially Expressed Genes (DEGs) for all groups. The 1500+ TempO-seq gene expression assay was used to determine differentially expressed genes compared to time-matched controls. DEGs were determined from 3-6 mice using a False Discovery Rate (FDR)-adjusted p-value of ≤0.05. All experimental groups were represented (see **Figure 1**), with the exception of S2-57 weeks, where samples were missing from the archive (NA, not available).

We determined differentially expressed genes (DEGs) of the treatment groups for all time points compared to the age-matched Controls Group (**Figure 2**; **Supplementary Data 1**). In general, the number of DEGs were greater the closer the measurement was to the DCA exposure window and then declined after DCA exposure was stopped regardless of the initial dosage duration. Groups with longer DCA exposures (S3 and Direct) had significantly more DEGs at 78-weeks compared to the shorter exposure durations (S1 and S2). This was similar to what was observed with select gene expression following DCA removal in our earlier study (23). We also observed a “peak” in DEGs for the timepoints measured following DCA removal for the S1 and S3 groups 22 and 21 weeks after exposure cessation, respectively. Archived samples were not available for S2-57 weeks group to confirm a similar peak in DEGs as that observed for the S1 and S3 groups. The majority of the DEGs were specific to the direct DCA exposure period, mouse age, and time after exposure. Only one gene, *Cavin2* (*Sdpr*), was differentially expressed in all timepoints. Some DEGS, such as *Irs1*, *Ctnnd1* and *Ebp* have the same expression trend over all timepoints but failed significance (FDR-corrected *p*-value <0.05) in some treatment groups. Others are altered in only specific treatment subgroups, such as *Col1a1*, *Col4a1* and *Cebpb* in S1 and S2 samples.

Ingenuity Pathway Analysis (IPA) was utilized to examine gene pathway enrichment for DEGs of the different treatment groups (**Supplementary Data 2**). In S1 and S2, early acute phase and inflammatory pathway signaling were upregulated initially, while many pathways linked to oxidative stress and known DCA effects were predicted to be downregulated (*sirtuin signaling, Hif1a signaling, unfolded protein response, oxidative phosphorylation*). At 78 weeks, enriched gene pathways were linked to long term cellular damage (*apelin liver signaling, wound healing signaling, intrinsic prothrombin activation, and GP6 signaling*), despite the relatively short exposure periods as compared to S3 and Direct groups. Enriched pathways differed for the S3 and Direct groups. Almost all significant pathways in the Direct group were predicted to be downregulated (*cholesterol biosynthesis, unfolded protein response, TGF-β signaling*). The S3 group contained multiple pathway alterations by 78 weeks, many of which were linked to predicted reduction in xenobiotic metabolism and enhanced signaling of crucial developmental and regeneration liver factors such as β-catenin and HIF1α. *NRF2 mediated oxidative stress response* was significantly down regulated in both S3 and Direct groups by 78 weeks. Of note, the activity of the lipid homeostasis regulator PPARα was predicted to be downregulated with early DCA exposure (S1-10) and up-regulated with later persistent DCA exposure (Direct group at 78 weeks; **Supplementary Figure S1**).

### DNA methylation alterations overview

**Figure 3:**
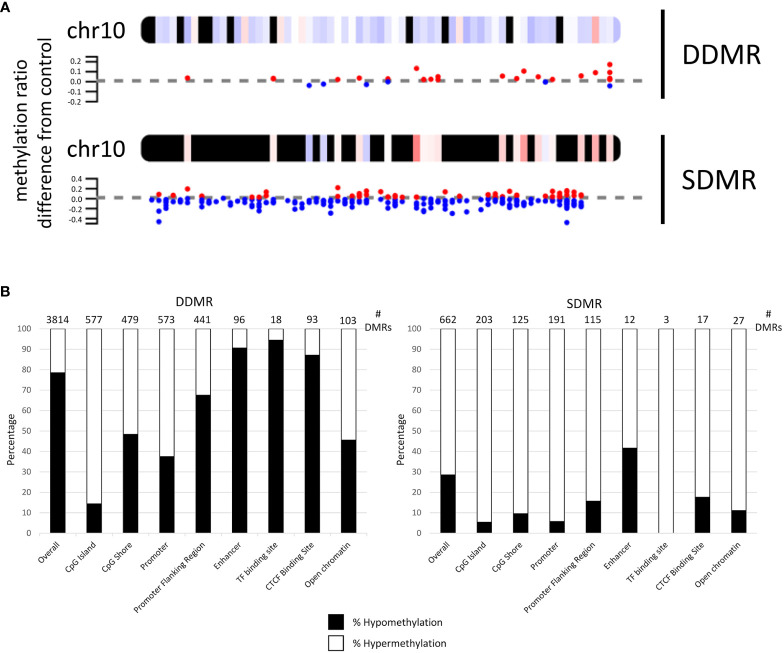
Mapping of differentially methylated regions for Direct (DDMR) and Stop (SDMR) 78-week measures. **(A)** Representative mapping DMRs for Chromosome 10. Red and blue dots represent hyper- and hypomethylated regions, respectively, as defined by calculations provided in *Methods*. Averaged intervals of hyper- or hypomethylation are represented by a gradient scale of red-to-blue, respectively, on the chromosome map. *Y-axis* represents the ratio of methylation differentiation compared to control. All chromosomal maps can be seen in **Supplementary Figure 2**. **(B)** DMR intersection with identified CpG and regulatory features in the mouse genome. Bar graphs represent the percentage of hyper- and hypo-methylated regions in different CpG and regulatory regions that are identified by Ensembl Regulatory Build (release 90; http://useast.ensembl.org/info/genome/funcgen/process/humanandmouse/annotation.html).

Annotation and characterization of differentially methylated regions (DMRs) are summarized in **Supplementary Data 3**. There were considerably more DMRs in the Direct group (DDMR; 3814 regions) compared to the Stop group (SDMR; 662 regions), likely attributed to diminishing effects of DCA with time away from exposure. This distinction was evident for all chromosomes (**Figure 3A**; **Supplementary Figure 2**). DMRs were mapped to predicted regulatory regions and CpG features in the mouse genome (**Figure 3B**). Although the overall numbers of DMRs were reduced in the Stop group samples, the percentage linked to promoters and CpG islands were roughly twice that of the DDMRs. Strikingly, the DDMRs are predominately hypomethylated (78.5% overall), while the SDMRs are predominantly hypermethylated (71.5% overall). Genes were linked to DMRs based on nearest transcriptional start site (TSS). With this characterization, 35% percent of the SDMR-linked genes intersected with those linked to DDMRs (**Supplementary Data 3**). Many members of major gene families that are dysregulated in liver disease (e.g., cancer) were linked to altered DNA methylation patterns in the DDMR group (**Table 1**) and partially in the SMDR group (bolded genes).

**Table 1:** Overview of major gene family differences in the DDMR as compared to controls.

Gene family function	Associated genes*
Extracellular Matrix Function and Regulation	*Adam*, ***Adamts*** *, Cdh*, ***Col***, ***Fgf*** *, Fndc, Sgcb*
Development, Metabolism and Cellular Regulation	***Adcy*** *, Ankrd*, ***Ccdc*** *, Cdk*, ***Dnah***, ***Fam***, ***Fbxl*** *, Fbxo*, ***Hox*** *, Klhl*, ***Lrrc***, ***Mrps***, ***Prr***, ***Rpl***, ***Sema***, ***Trim***, ***Usp***, ***Wnt***, ***Zdhhc***
GCPR and Rho GTPase Cell Signaling Activity	*Adgrl*, ***Arhgap*** *, Arhgef, Dock*, ***Gpr***, ***Rab***, ***Rgs***
Transmembrane Signaling and Transport	***Atp*** *, Cacna*, ***Epha*** *, Galnt, Kcna, Kctd*, ***Kif***, ***Pcdh***, ***Rnf*** *, Robo*, ***Slc*** *, Slitrk*, ***Syt***, ***Tmem***
Gene Regulation	*Ddx*, ***Fox***, ***Parp***, ***Prdm*** *, Snord, Wdr*, ***Zfp***, ***Zscan***

* Bolded genes are also associated in the SDMR.

**Figure 4:**
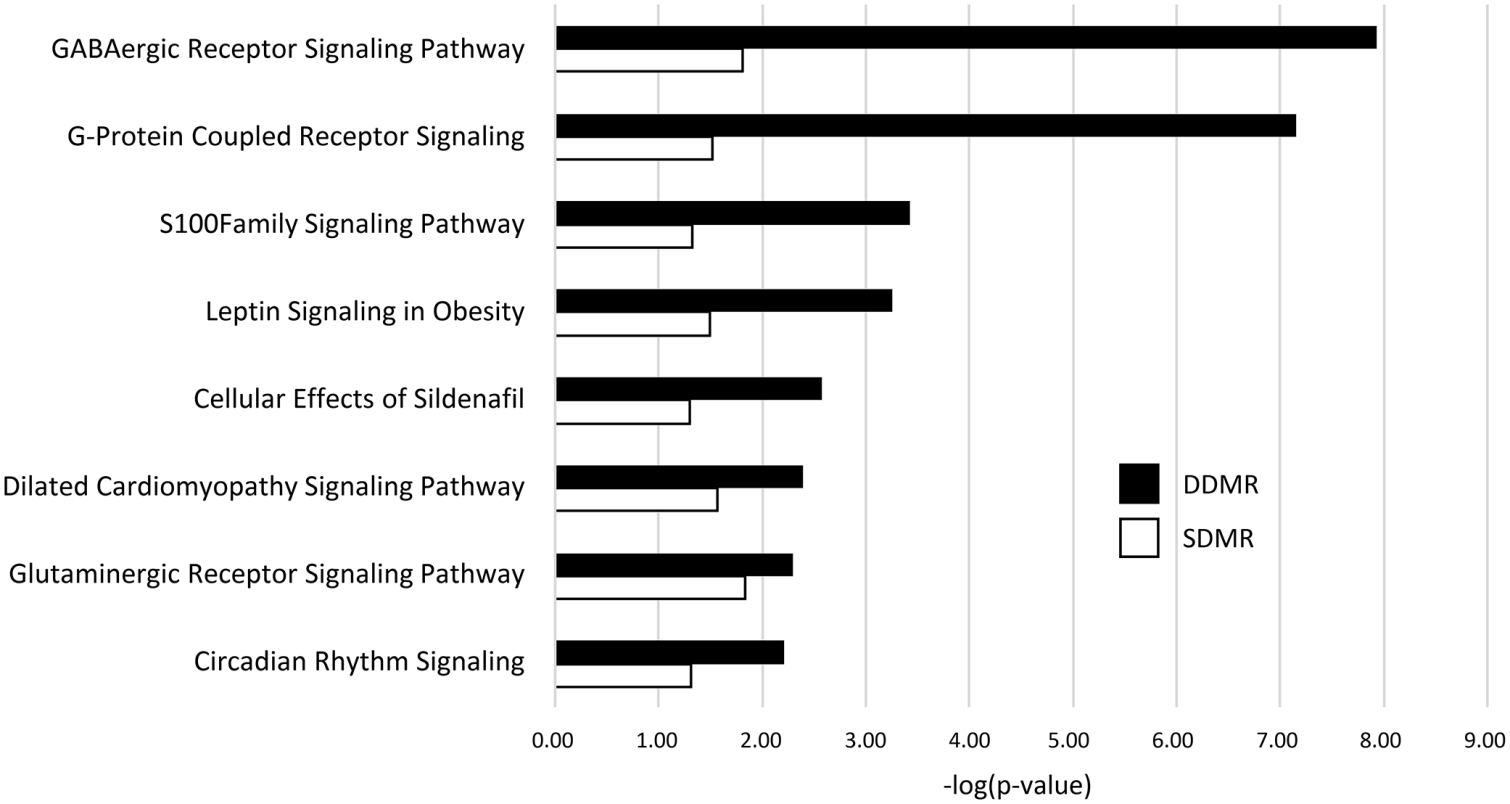
DMR-linked genes enrichment of canonical gene pathways. Genes linked to DMRs in both the Direct (DDMR) and Stop (SDMR) groups by mapping to the nearest Transcriptional Start Site (TSS). All gene pathways that were enriched in both groups are graphed. Enrichment was considered significant if Fisher’s Exact test -log (*p*-value) was greater than 1.3.

Related, gene pathway analysis using IPA indicated 101 significant pathways were enriched with DDMR-linked genes, while only 15 were enriched with genes linked to SDMR (**Supplementary Data 4**). Eight of the 15 SDMR-linked gene pathways (53%) intersected with DDMR-linked pathways (**Figure 4**). These intersecting pathways are involved with regulation of cellular physiology and metabolism, such as G-protein coupled receptor and S100 family signaling pathways. Interestingly, we observed enrichment of the neurotransmitter-linked signaling pathways, GABAergic receptor and glutaminergic receptor signaling pathways.

### Time course analysis of continuous and persistent effects of DCA exposure

**Figure 5:**
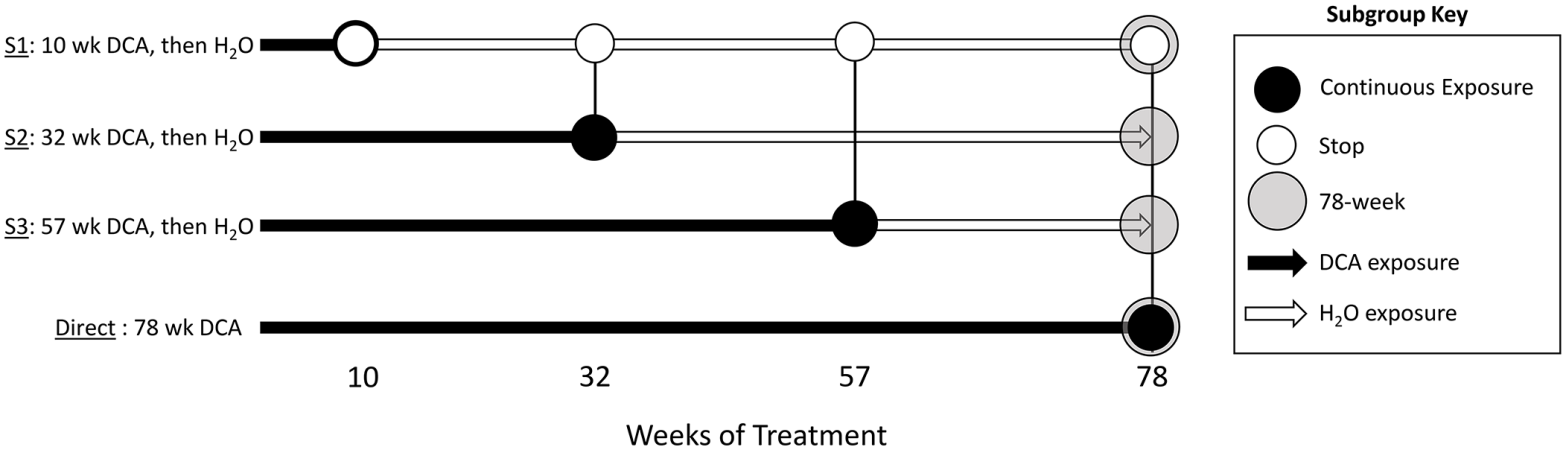
Overview of subgrouping of study timepoints. We defined the “Continuous Exposure” subgroup as samples from mice that were continuously exposed to DCA (S1-10, S2-32, S3-57, and Direct-78; black-filled dots. The “Stop” subgroup consisted of samples collected from mice exposed to 10 weeks of DCA only at all collected timepoints (the S1 group progression: S1-10, S1-32, S1-57, and S1-78; white dots). The “78-week” subgroup consisted of all the samples collected at 78 weeks (S1-78, S2-78, S3-78, and Direct-78; grey-filled dots).

To explore age and treatment interactions for both continuous and previous DCA exposures, we divided the analysis into three subgroups (**Figure 5**). First, we grouped all the timepoints collected while the animal was continuously exposed to DCA (S1-10, S2-32, S3-57, and Direct-78), referred to as the “Continuous Exposure” subgroup. The second subgroup analyzed the persistent effects of DCA after 10 weeks of exposure before stoppage (the S1 group progression: S1-10, S1-32, S1-57, and S1-78), referred to as the “Stop” subgroup. The last subgroup, “78-week”, consisted of all the 78-week timepoints (S1-78, S2-78, S3-78, and Direct-78) and is most representative of the cumulative DCA mediated changes over time for both continuous and persistent effects. DMR-linked genes as part of these subgroup comparisons and a master table of all DEG and DMR-linked gene intersects, including gene pathway associations, are listed in **Supplementary Data 5**.

### Continuous exposure subgroup analysis

**Figure 6:**
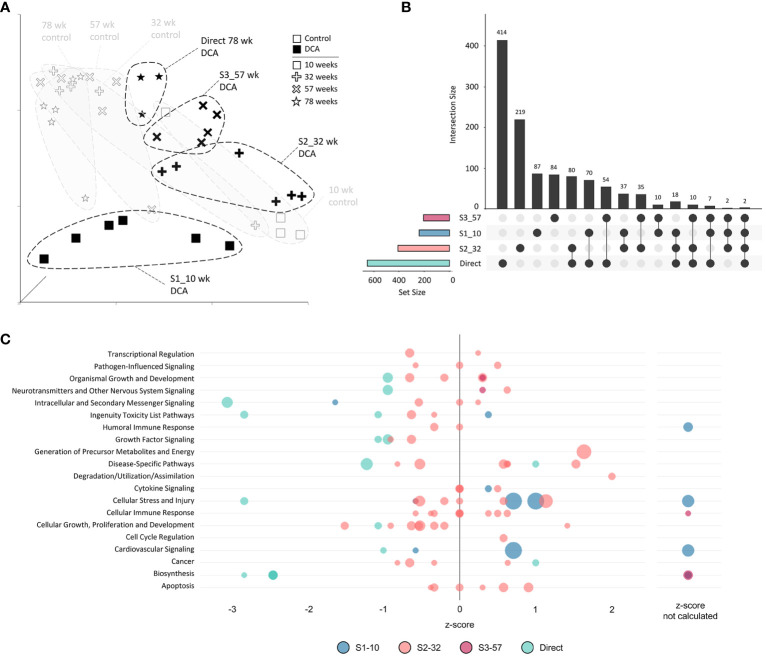
Continuous exposure subgroup differentially expressed genes and enriched canonical gene pathways. **(A)** Uniform Manifold Approximation and Projection (UMAP) for the “Continuous Exposure” subgroup samples. UMAP is based on 15 Principal Components of normalized count data before DEG determination, which captured over 99% of the variation displayed in these samples. **(B)** UpSet plot demonstrating differences and similarities of significantly (FDR-corrected *p*-value<0.05) altered genes compared to time-matched controls. Connected dots identify the group(s) that match to the adjacent bar and DEG number unique for those samples. **(C)** Bubble graph representing gene expression pathways enriched by the “Continuous Exposure” subgroup. Each bubble represents an individual canonical pathway, calculated by Ingenuity Pathway Analysis (IPA), that are categorized by larger meta-categories along the y-axis. The right-to-left placement identifies predicted pathway activation or repression (positive or negative z-score, respectively). Some pathways did not have the data to generate this prediction (no z-score). The size represents the -log(*p*-value) of the pathway enrichment. The color indicates the experimental mouse group that the pathway enrichment was measured.

The unique nature of each timepoint for both controls and continuous DCA treatments was demonstrated by UMAP (**Figure 6A**) with the most divergent groups seen early at 10 weeks, both with and without DCA exposure. Approximately half or more DEGs induced by DCA exposure were unique to a particular timepoint (**Figure 6B**). Only 2 DEGs were shared across all four timepoints (*C9*, *Sdpr*), which increased to 18 DEGs when comparing timepoints S1-10, S2-32, and 78-week (Direct). DEG intersections between 10-/32- and 78-week (Direct) timepoints were 70 and 80 DEGs, respectively. When comparing shared DEGs between the Direct group and S1-10, S2-32, or S3-57, the percentages were 48%, 43%, or 45%, respectively, demonstrating some degree of commonality between earlier and later DCA exposure related effects. Strikingly, most of the DEGs shared between S1-10 and 78-week (Direct) timepoints demonstrated opposite expression patterns suggesting an important age interaction with continuous DCA exposure. This “flip” in directionality is also demonstrated at a gene pathway level, where S1-10 pathways are primarily activated, and 78-week (Direct) pathways repressed within or across pathway categories (**Figure 6C**). The intermediate timepoints (S2-32 and S3-57) showed a mixture of predicted activation and repression states. The cellular stress and injury pathways were a clear example of this drift over time, suggesting a switch from acute stress response to liver dysfunction and cancer.

**Figure 7:**
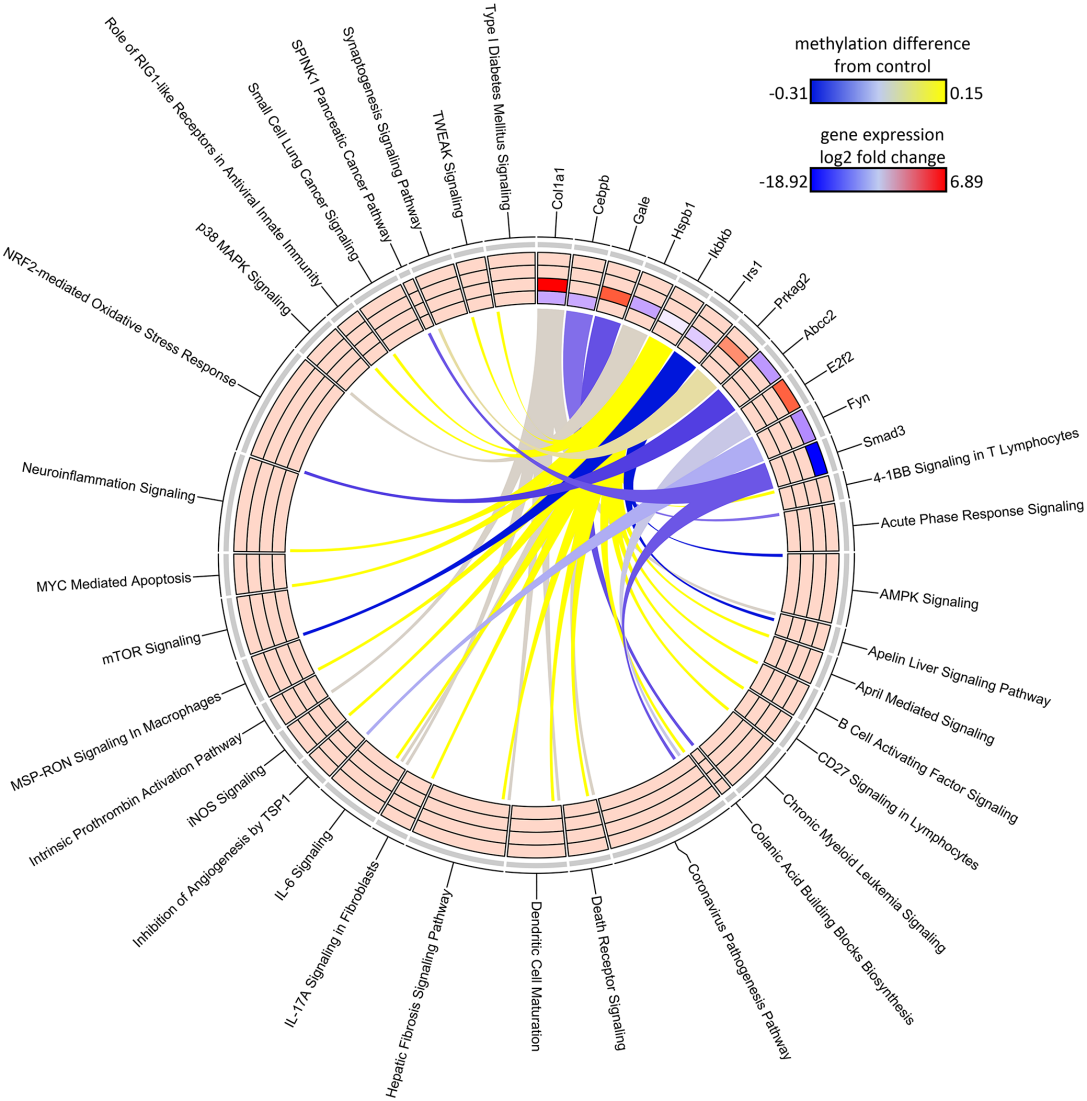
Circos plot of DDMR genes linked to “direct” group gene pathways. Connections denote DDMR-linked genes with canonical gene pathways that are enriched by DEGs identified in the Direct groups. Color gradient of connections denote degree of methylation change compared to control mice (yellow = hypermethylated, purple = hypomethylated). Tracks denote log_2_ fold-change (red = upregulation, blue = downregulation) of gene expression compared to time-matched controls for S1 10-week, S2 32-week, S3 57-week and Direct 78-week sample groups (inside -> outside). Slice size for individual pathways is proportional to number of DEGs that link to each individual pathway.

The DNA methylation assessment at 78 weeks may not only reflect the current gene regulatory state, but also previous perturbations to the epigenome at earlier timepoints. Due to lack of archived samples available for this study, we could only assess DNA methylation at 78 weeks and, instead, used the DEGs measured at all the collection time points as a proxy for gene activity that may be influenced by epigenetic state. Given DEGs from the Continuous Exposure subgroup were derived from mice in which DCA exposure was not removed, the DDMR data were the best comparator. The overall number of DEGs intersecting with DDMR-linked genes (as defined most loosely by the closest gene TSS only or more stringently by further refining DDMRs also located within predicted gene regulatory regions) were low (**Table 2**). Despite these low intersections, we observed DEGs at all timepoints reflected in the DDMR-linked gene list. When combined across timepoints, 11 DDMR-linked genes (TSS and regulatory region defined) overlapped with DEGs in the Continuous Exposure subgroup that were also part of significant gene pathways at the collected timepoints (**Figure 7**, **Table 2** last column). These genes were included in pathways mediating inflammation, oxidative stress response, and mitotic signaling/regulation, with many downregulated and linked to hypomethylation at 78 weeks. Because we observed this overlap in DDMR-liked genes and DEGs that play roles in pathways known to be perturbed by DCA exposure in liver, this suggested that DDMRs present at 78 weeks reflected gene regulatory patterns observed, at least in part, by earlier and current DCA exposure.

**Table 2:** Continuous exposure subgroup and DMR-linked genes intersection.

	DEGs	TSS-only Defined		TSS and Reg Region Defined	
		DEG/DDMR Gene Intersect	DEG/DDMR Intersected Genes in Pathways	DEG/DDMR Gene Intersect	DEG/DDMR Intersected Genes in Pathways
**S1-10**	233	23	3	16	2
**S2-32**	403	53	16	25	5
**S3-57**	204	15	1	11	1
**Direct**	655	53	8	28	4

### Stop subgroup analysis

**Figure 8:**
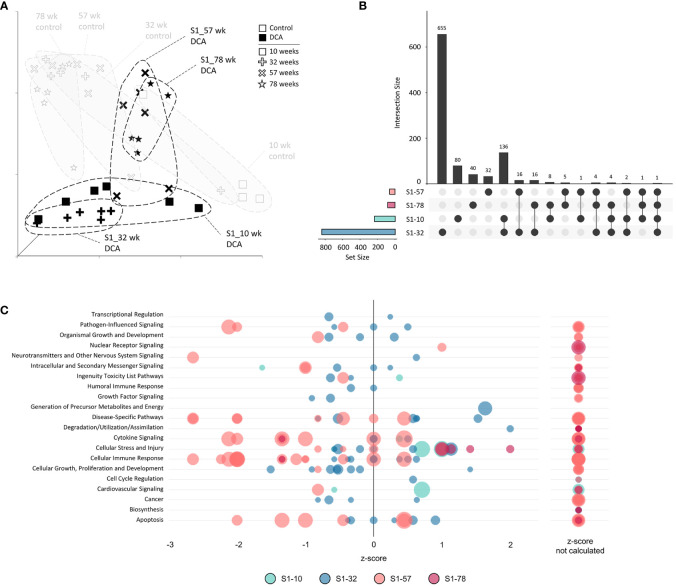
Stop subgroup differentially expressed genes and enriched canonical gene pathways. **(A)** Uniform Manifold Approximation and Projection (UMAP) for the “Stop” subgroup samples. UMAP is based on 15 Principal Components of normalized count data before DEG determination, which captured over 99% of the variation displayed in these samples. **(B)** UpSet plot demonstrating differences and similarities of significantly (FDR-corrected *p*-value<0.05) altered genes compared to time-matched controls. Dots identify the group(s) that match to the adjacent bar and DEG number unique for those samples. **(C)** Bubble graph representing gene expression pathways enriched by the “Stop” subgroup. Each bubble represents an individual canonical pathway, calculated by Ingenuity Pathway Analysis (IPA), that are categorized by larger meta-categories along the y-axis. The right-to-left placement identifies predicted pathway activation or repression (positive or negative z-score, respectively). Some pathways did not have the data to generate this prediction (no z-score). The size represents the -log(*p*-value) of the pathway enrichment. The color indicates the experimental mouse group that the pathway enrichment was measured.

We next examined the gene expression persistence and DNA methylation after only 10 weeks of treatment to better understand the carryover effects of early DCA exposure. The UMAP visualization summary of gene expression across the S1 Stop group demonstrated the similarity of the 10- and 32-week timepoints and clear separation to later timepoints and age-matched controls (**Figure 8A**). The data indicated that S1-57 and S1-78 samples grouped closer to controls, however, separation is still evident indicating persistent gene expression effects well after cessation of exposure. As mentioned earlier, S1-10 and S1-32 DEGs were more numerous than the later S1-57 and S1-78 group DEGs. The largest DEG intersection was between the S1-10 and S1-32 groups (136 genes), where 58% of S1-10 DEGs were shared (**Figure 8B**). At a pathway level, S1-10 and S1-32 groups also tended to enrich similar categories, in particular cell stress and injury and toxicity pathways (**Figure 8C**). Pathways associated with S1-57 and S1-78 tended to be repressed, with the notable exception of activated cell stress and injury pathways at 78 weeks. Conversely, related pathways of immune response and cytokine signaling tended to drift from an activated state to a repressed state over time.

**Figure 9:**
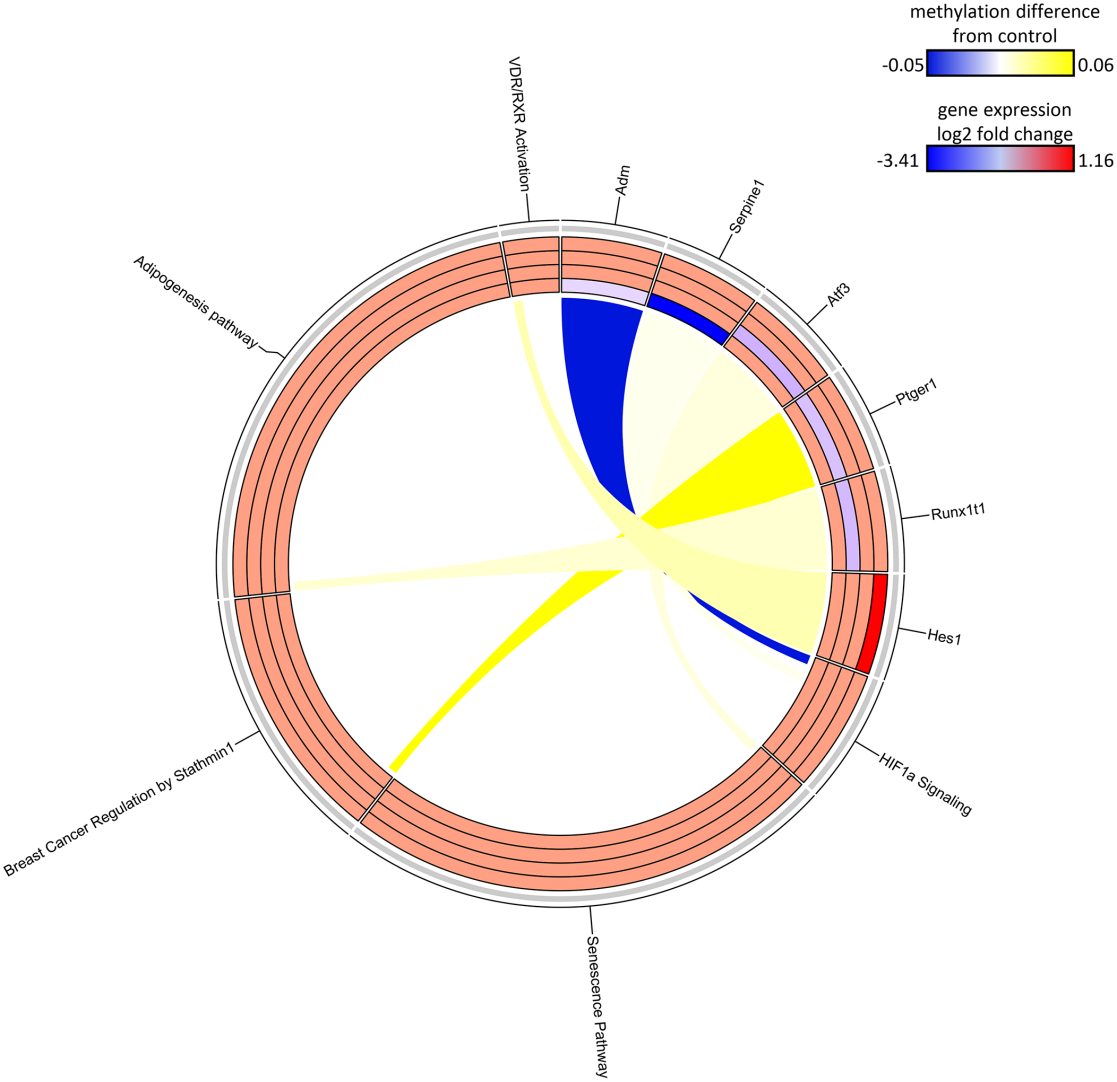
Circos plot of SDMR genes linked to “stop” subgroup gene pathways. Connections denote SDMR-linked genes with canonical gene pathways that are enriched by DEGs identified in the S1 groups. Color gradient of connections denote degree of methylation change compared to control mice (yellow = hypermethylated, purple = hypomethylated). Tracks denote log_2_ fold-change (red = upregulation, blue = downregulation) of gene expression compared to time-matched controls for S1 10-week, S1 32-week, S1 57-week and S1 78-week experimental groups (inside -> outside). Slice size for individual pathways is proportional to number of DEGs that link to each individual pathway.

The SDMR methylation measures were derived from the S1-78 sample group, and similar to the DDMR analysis for the Direct group comparisons, we believed the SDMR data could reflect both the epigenomic state of the matched timepoint and persistent alterations from earlier time points. To assess this, we examined the intersect of S1 Stop subgroup DEGs and SDMR-linked genes as defined by TSS only or by TSS and proximity to mouse gene regulatory regions at all time points (**Table 3**). Overall numbers of intersects were low, due to the limited number of total SDMR-linked genes, but those shared genes that mapped to pathways tended to demonstrate downregulation at earlier timepoints and upregulation at the 78-week timepoint (**Figure 9**). Downregulated genes were primarily hypermethylated in S1-10 and S1-32 treatment groups and have roles in HIF1a signaling, senescence, cancer, and adipogenesis. The upregulated gene *(Hes1*) at S1-78 was hypermethylated and involved in the *Vitamin D Receptor/RXR activation* pathway.

**Table 3:** Stop group DEGs and DMR-linked genes intersection.

	DEGs	TSS-only Defined		TSS and Reg Region Defined	
		DEG/SDMR Gene Intersect	DEG/SDMR Intersected Genes in Pathways	DEG/SDMR Gene Intersect	DEG/SDMR Intersected Genes in Pathways
**S1-10**	233	8	2	7	2
**S1-32**	834	17	6	9	3
**S1-57**	62	0	0	0	0
**S1-78**	79	2	1	1	1

### 78-week group timepoint analysis

**Figure 10:**
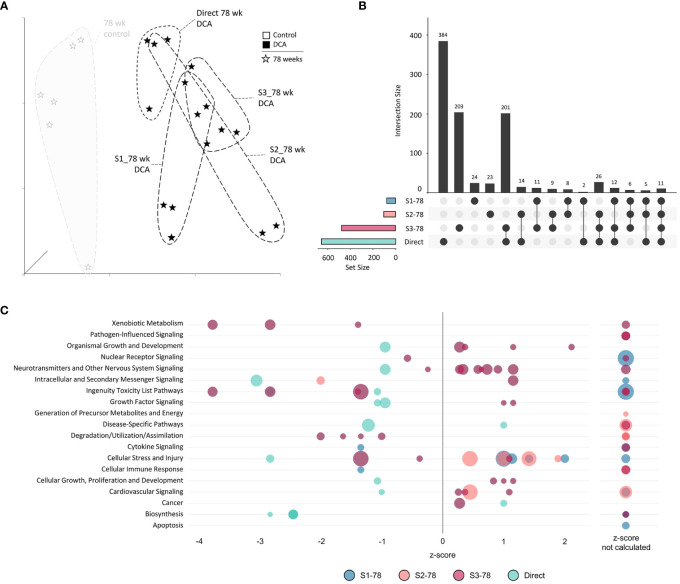
78-week subgroup differentially expressed genes and enriched canonical gene pathways. **(A)** Uniform Manifold Approximation and Projection (UMAP) for “78-week” subgroup samples. UMAP is based on 15 Principal Components of normalized count data before DEG determination, which captured over 99% of the variation displayed in these samples. **(B)** UpSet plot demonstrating differences and similarities of significantly (FDR-corrected *p*-value<0.05) altered genes compared to time-matched controls. Dots identify the group(s) that match to the adjacent bar and DEG number unique for those samples. **(C)** Bubble graph representing gene expression pathways enriched by the “78-week” subroup. Each bubble represents an individual canonical pathway, calculated by Ingenuity Pathway Analysis (IPA), that are categorized by larger meta-categories along the y-axis. The right-to-left placement identifies predicted pathway activation or repression (positive or negative z-score, respectively). Some pathways did not have the data to generate this prediction (no z-score). The size represents the -log(*p*-value) of the pathway enrichment. The color indicates the experimental mouse group that the pathway enrichment was measured.

Finally, we assessed if varying DCA exposure durations altered the gene expression response at 78 weeks by comparing all the 78-week sample DEGs. UMAP visualization of the 78-week subgroup DEGs demonstrated separation of treatment groups compared to the 78-week aged control (**Figure 10A**). S1-78 and S2-78 groups demonstrated higher variability among individual samples, compared to the S3-78 and Direct-78 week, which exhibited the most similarity to each other. This was also reflected in the degree of DEGs overlap between the later timepoints (S3-78 and Direct treatment groups; 201 genes, **Figure 10B**), possibly reflecting the continuum of changes due to lengthy DCA exposure with age and metabolic status. As noted previously, DEGs are greatly reduced in the S1-78 and S2-78 groups, likely due to *near* return to cellular homeostasis over time after DCA exposure. Of note, 70% and 77% of the S1-78 and S2-78 DEGs, respectively, intersected one or more longer exposed samples, indicating persistent DEGs are similar to responses observed in more continuous exposures. Gene pathways linked to the Direct and S3-78 treatment groups are primarily repressed, especially for continuous DCA exposure (Direct 78-week group), and linked to toxicity, cell stress and injury, and metabolism (**Figure 10C**). Pathway enrichment analysis of S3-78 DEGs indicated activation of neurotransmitter signaling and cellular growth, where both S3-78 and Direct groups demonstrated activation of cancer pathways. Pathway enrichment of S1-78 and S2-78 DEGs suggested activation of cellular stress and repression of immune pathway categories.

While genes linked to DDMRs and SDMRs were respectively derived from Direct 78-week and S1-78-week samples, we assessed if there were any commonalities with other 78-week samples. By percentage, the S1-78 and S2-78 group DEGs, which represented earlier-in-life exposures and longest periods of persistent gene alterations, demonstrated the larger overlap to DDMR-linked genes (**Table 4**). Specifically, 8.9% (7 of 79) of S1-78 and 8.8% (9 of 102) of S2-78 intersected DEGs with DDMR-linked genes (defined by proximity to TSS and also within a gene regulatory region). This is compared to 5.0% (24 of 476) in S3-78 and only 4.3% (28 of 655) in the Direct 78-week group. These overlapped DEGs also demonstrated a larger proportion mapping to significant canonical pathways in the sample groups with longer time since DCA exposure compared to group with more recent exposure (**Table 4**, *last column, DDMR rows*), providing some evidence that these genes linking to direct DCA-mediated methylation changes are known components of pertinent gene pathways that persisted well after DCA exposure. As previously noted, few S1-78 DEGs intersected with SDMR-linked genes. A greater number of DEGs of the longer DCA exposures overlapped SDMR-linked genes, but this is likely due to chance because of the larger overall DEGs with the samples of more recent DCA exposures.

**Table 4:** 78-week groups DEGs and DDMR/SDMR-linked genes intersection.

	DEGs	TSS-only Defined		TSS and Reg Region Defined		
		DEG/DMR Gene Intersect	DEG/DMR Intersected Genes in Pathways	DEG/DMR Gene Intersect	DEG/DMR Intersected Genes in Pathways	
**S1-78**	79	14	10	7	6	DDMR
**S2-78**	102	14	7	9	5	
**S3-78**	476	41	17	24	11	
**Direct**	655	53	8	28	4	
**S1-78**	79	2	1	1	1	SDMR
**S2-78**	102	1	0	1	0	
**S3-78**	476	7	2	5	1	
**Direct**	655	9	0	5	0	

## Discussion

Persistent effects of chemical exposures that reprogram the metabolic profile of cells can have important impacts later in life. In liver tumors, cancer cells favor glycolysis versus oxidative phosphorylation even in the presence of oxygen, a process known as aerobic glycolysis or the Warburg effect (31). This cancer-mediated metabolic programming is thought to maintain an ideal environment for tumor initiation and growth by reducing mitochondrial-derived reactive oxygen species (ROS) production and apoptotic pathway activation, maintaining the genomic stability of stem cells, and maintaining a favorable acidic microenvironment through lactate production (32–34). DCA, a pyruvate analogue, shunts metabolism toward oxidative phosphorylation thereby promoting ROS production, anti-growth signaling and apoptosis through mitochondrial cytochrome c release (35–37). Because of this metabolic reprogramming that appears to counteract the Warburg effect of cancer cells, DCA and other small molecule PDK inhibitors have been extensively studied as a cancer (and other metabolic disease) therapeutic (15). However, persistent effects due to early-in-life exposure to DCA instead *increased* tumor incidence compared to controls in mouse liver (1, 21–23) and the causal mechanism has not been fully characterized. Using gene expression and DNA methylation profiles of archived samples from a previously characterized stop exposure time-course study (22, 23), we further examined the underlying mechanisms of continuous and carryover effects of DCA in mouse liver. First, we observed significant gene expression changes linked to metabolic reprogramming with continuous DCA exposure; however, these expected patterns changed with age and transitioned to a pro-tumorigenic characterization. Second, carryover effects of DCA followed a different trajectory than continuous, 78-week DCA exposure, but also was heavily influenced by age. Finally, DNA methylation alterations measured at 78-weeks indicated both continuous and carryover effects of DCA exposure.

The tumorigenicity of DCA in rodent liver has been studied for nearly 30 years and evidence has stipulated that continuous higher exposures (*e.g.*, ≥3.5g/L in drinking water) can lead to overt hepatoxicity via disruption of normal cellular metabolism (9, 13) which could have a relationship with cellular regeneration but, there has not been a clear consensus on carcinogenic mode-of-action for direct DCA exposure (38). Our gene expression analysis recapitulated previous transcriptomic findings in many cases; the inclusion of time-matched controls over all measured time points also supported progression of phenotypic observations. By 32 weeks on continuous DCA exposure, gene expression alterations predictively enriched pathways involving activation of oxidative phosphorylation (*TCA cycle II*, *methionine degradation*), increased apoptosis signaling (*p53*, *ATM, death receptor*, *MYC signaling*), and decreased cell growth signaling and increased quiescence (*TGF-β*, *mTOR/AMPK*, *Id1*). We also observed upregulation of many Serpin family members (*e.g.*, *SerpinA3*, *SerpinD1*) and the Complement system (*e.g.*, *C9*) (39, 40) after 10 weeks of continuous DCA exposure that subsequently fell below control levels by 78 weeks, which matched the transient mild to moderate inflammation with early exposure (23). Interestingly, at the later timepoints of continuous DCA exposure, we observed increased proliferative signaling such as the *D-myo-inositol (1,4,5)-trisphosphate biosynthesis* pathway at 57-weeks and augmentation of the peroxisome proliferator-activated receptor alpha pathway (PPARαby 78-weeks, which flipped from a suppression of the PPARα pathway at 10-weeks of DCA exposure (**Supplementary Figure 1**). This activation was matched with inhibited STAT5b signaling, which has been noted mutually antagonistic to PPAR and other xenobiotic receptor activation and is characterized by “feminization” of the liver due to decreased growth hormone (GH) secretion leading to a profile more common in female versus male rodents (41). GH secretion can be suppressed through an increased amount of circulating glucose levels such as during periods of increased gluconeogenesis (42); the converse is seen with high-dose DCA exposure (43). While we observed robust PPARα target gene induction previously with DCA treatment after 6 days (23), general consensus is DCA does not lead to liver cancer through a PPAR-mediated pathway (44). However, increased hepatic peroxisome proliferation activity was observed in mice with the dose used in this study, which equated to ~429 mg/kg-day (13, 23). This overall shift away from early DCA-like responses may reflect later processes initiated by early tumor formation at 78 weeks, which may be a result of DCA-induced hepatotoxicity with constant exposure, leading to secondary effects of regenerative proliferation and/or receptor-mediated mitogenesis.

DCA augments tumorigenesis even if the exposure window is limited to early life. Greater than 85% of the tumorigenic effect is captured with 10-weeks of exposure compared to a lifetime exposure at the same 3.5 g/L dose (22). The mechanisms of these persistent effects of DCA after short-term exposure are more uncertain than those of continuous exposure. Phenotypically, the most lingering effects in the liver included increased liver weight and hepatocellular hypertrophy, which resolved by 26 weeks after DCA removal. Liver weight again increased later in the study at 93 weeks likely due to the presence of tumors. Hepatocellular necrosis and mild inflammation also transiently persisted but resolved more quickly by 5 weeks after DCA removal (23). The effects were attributed to increased glycogenosis and glycogen storage which is an established hallmark in hepatocytes with high DCA exposure (9, 43) and may have contributed to acute hepatotoxic response during the exposure period. In our gene expression measurements, the two timepoints assessed after DCA removal (S1-32 and S3-78 weeks) exhibited a transcriptional burst of activity, with 155 DEGs in common, suggesting a similar response to the removal and return to homeostasis after DCA removal. Intriguingly, 70% of these genes exhibited opposite regulation between the two points, where the earlier 32-week timepoint favored known DCA effects of increased apoptosis, inflammation and suppressed growth signaling but, the converse was observed at 78-weeks. This is consistent with the phenotypic effects we previously measured, where mild necrosis and some inflammation, were observed at earlier ages with current DCA exposure, but were not observed with DCA exposure later in life (≥78 weeks) with current exposure (23). This suggested that the aged mouse liver responds differently to the effects of DCA. To further support that age heavily influenced cellular effects of direct or immediate post-DCA exposure, we observed similar overlap of 281 DEGs between the direct DCA exposed mice (S2-32) and recently exposed (S1-32), both samples of which were taken at 32 weeks. All but one of these genes shared the same directionality compared to controls and, again, linked to hallmark responses of DCA exposure. Therefore, immediate transcriptional carryover effects of DCA were robust, but mouse age heavily influenced the type of response much like what we observed in the direct only exposures: from DCA-like early to more cancer-like later.

Our results showed the transcriptional response to DCA in groups furthest out from DCA exposure (e.g., S1-78, S2-78) were greatly reduced compared to those mice closer to DCA exposure windows, but some signal did persist to the 78-week timepoint. The DEGs and gene pathways were similar between these two groups (100% of the shared DEGs displayed the same directionality) and were well represented by upregulated collagen genes that enriched pathways of apelin signaling and mechanisms that regulate fibrogenesis. Apelin is an adipokine that is the ligand for the G protein-couple receptor APJ (45) and has been of focus due to its sensitivity to glucose homeostasis and link to diabetes and insulin resistance (46). In liver, apelin exposure also promotes glycogenosis through the insulin signaling pathway (47), suggesting this response may be latent activity of DCA although hallmarks of excessive hepatic glycogen storage are no longer present at this point (23). Curiously, apelin is linked to liver fibrogenesis and mediates the expression of mesenchymal markers such as collagen (48–50), which are upregulated gene features at 78 weeks in the samples >26 weeks removed from DCA exposure. Conversely, a DCA derivative was linked to anti-fibrotic activity in hepatic stellate cells (HSCs) by suppressing the mesenchymal transition of these cells, presumably through anti-glycolytic effects of this compound (51, 52). This reflected the anti-fibrotic gene signature noted at 10-weeks of DCA exposure in our study (*e.g*., reduced *HIF1α* pathway, reduced collagen gene expression). The persistent effects of DCA, which were more similar to results we observed with continuous DCA exposure later in life, reflected cellular processes that opposed the known effects of early, acute DCA exposure. This pattern, again, underscores an important interaction of DCA-mediated effects in the mouse liver and aging.

Commonality between the continuous and persistent effects of DCA exposure may be reflected in the DNA methylation profiles. Like gene expression, the methylation differences in the mice with 10-week, early-life DCA exposure were less robust than mice with continuous exposure by 78-weeks. Despite these differences, the altered methylation linked to gene pathways such as cellular signaling, regulation and metabolism shared in both the continuously exposed and the Stop groups. For example, we saw enrichment of genes linked to the gamma-aminobutyric acid (GABA) and glutaminergic signaling pathways in this intersection. The methylation profiles of these specific genes matched in the Direct and Stop samples, despite the general trend of hypomethylation in the Direct samples (also noted previously in female B6C3F1 mice [53)] and hypermethylation in the Stop samples. While commonly known as a neurotransmitter in the brain, GABA (a glutamate metabolite), can have effects in other metabolic organs including the liver (54). Hepatic GABA signaling is involved in mechanisms such as glucoregulation via insulin signaling and membrane transport, oxidative stress damage, and lipid metabolism (55–57). Gene pathways altered in our study that reflected such mechanisms were found in continuous DCA exposures (*e.g.*, *Nrf2 oxidative stress*), persistent effects (*e.g*., *insulin pathway signaling*), and both (*e.g*., *cholesterol biosynthesis*), and were modulated by age and exposure length. Since DCA exposure increases glutamate production (58) and GABA-transaminase activity is increased through the TCA cycle which mediates GABA synthesis (54), likely there is an increase in GABA availability after early DCA exposure, impacting a variety of pathways, and leaving an indelible mark in the methylome.

We observed that genes linked to methylation changes after continuous exposure primarily overlapped DEGs at early time points for both the continuous and short-term DCA exposed mice, suggesting the aged methylome may reflect earlier events of DCA effects. The interrogation of methylation changes in the epigenome is increasingly seen in the context of lifetime and transgenerational assessments in human health. The seminal studies in the agouti mouse model demonstrated that prenatal nutritional supplements altered DNA methylation in the offspring with lasting phenotypic results into adulthood (59, 60). Similarly, the 2008 “Dutch Hunger Winter” study demonstrated that pregnancy during famine conditions led to increased adverse health outcomes in adult offspring, due to the methylation change in a single locus (61). Since that time, many environmental exposures have been examined in human and model population studies for methylation and disease state correlations (62, 63). Ongoing research in the field has reinforced the correlation between early life exposure and later life disease progression. Trevino et al. used a rat model study with an early life endocrine disrupting chemical and showed that early life epigenomic reprogramming led to adult metabolic disruption which was not evident until a later life dietary change (64). Li et al. examined lifetime methylation changes in a twin study assessing various physiological and health related parameters (65). They demonstrated that methylation changes in the epigenome, while most highly linked by genetic factors and gene regulation, are also strongly linked to cohabitation status, indicating the lifetime influence of early life environmental factors in adult growth, development, and likely disease progression. These methylation changes can either be persistent or transient once the environmental stressor is removed, leading to biomarkers that can indicate past or cumulative exposure or the “exposome” (66, 67).

## Conclusion

Here, we measured the transcriptomic and epigenomic alterations due to increasing windows of exposure of the metabolic reprogramming chemical DCA in the male B6C3F1 mouse model. Previous studies were inconclusive about the mechanism of latent tumorigenic effects of DCA, not supporting classical routes such as genotoxicity, chronic oxidative stress, regenerative proliferation, and cytotoxicity. Using modern methods to assess ‘omic-based measurements in archived formalin-fixed mice liver samples, we observed that responses due to DCA exposures early in life differed greatly than those later in life. In general, anticipated anti-Warburg related DCA effects were measured early, while opposing, more pro-tumorigenic pathways were noted later in life. Additionally, the length of time of DCA exposure impacted the robustness of response, but still followed a cancer-like “switch” which was dependent on mouse age. DNA methylation patterns at 78-weeks reflected early-life alterations in genes and pathways and likely impacted regulatory effects later in life. These data, along with previous studies, suggests that persistent metabolic shifts, whether they be due to consistent DCA exposure or persist through epigenetic means, interact with normal aging mechanisms to result in pro-tumorigenic environment. The impact of early-life, non-genotoxic exposures on later cancer outcomes is a major challenge for risk assessment when considering protective thresholds in the environment. By deriving the mechanistic basis for a chemical mode-of-action for latent carcinogenic effects in model systems, additional weight of evidence can be factored for transient exposures rather than relying on chronic life-time exposures in standard bioassays (1). In addition, biomarkers derived from these studies can be utilized to assist with chemical hazard identification where there is limited information, or where more non-classical tumorigenic modes-of-action, such as epigenetics, are occurring.

## Data availability statement

The original contributions presented in the study are included in the article/**Supplementary Material** and underlying data for all figures can be accessed at DOI: 10.23719/1529544. Sequencing data is available NCBI/Gene Expression Omnibus (https://www.ncbi.nlm.nih.gov/geo/) under accession #GSE242665. Further inquiries can be directed to the corresponding author.

## Ethics statement

The animal study was approved by EPA Institutional Animal Care and Use Committee. This study used archived samples from this original study. The study was conducted in accordance with the local legislation and institutional requirements.

## Author contributions

GC: Data curation, Formal analysis, Investigation, Methodology, Validation, Visualization, Writing – original draft, Writing – review & editing. JC: Data curation, Formal analysis, Methodology, Software, Writing – original draft, Writing – review & editing. BB: Data curation, Formal analysis, Methodology, Software, Writing – original draft, Writing – review & editing. PB: Formal analysis, Methodology, Resources, Supervision, Writing – original draft, Writing – review & editing. BC: Conceptualization, Data curation, Formal analysis, Funding acquisition, Investigation, Methodology, Project administration, Resources, Supervision, Visualization, Writing – original draft, Writing – review & editing.

## Supplementary material

Supplemental Data 1 Complete DEG lists for all sample groups.

Supplemental Data 2 IPA Canonical Pathway Analyses for DEGs in all sample groups.

Supplemental Data 3 DMR locations and annotations for both Direct and Stop groups at 78 weeks.

Supplemental Data 4 IPA Canonical Pathway Analyses of Genes Linked to DMR Regions Defined by Closest Transcriptional Start Site (TSS) for Both Direct (DDMR) and Stop (SDMR) Groups.

Supplemental Data 5 DMR-linked Genes Defined by Closest Transcriptional Start Site (TSS) for Both Direct (DDMR) and Stop (SDMR) Groups that Intersect DEGs and Associated Canonical Pathways for All Timepoints.

Supplementary Figure 1 Ingenuity Pathway Analysis (IPA) *de novo* gene network generation based on DEGs from continuous 3.5 g/L DCA exposure in drinking water at 10 and 78 weeks. Arrows and capped lines represent known upregulation and downregulation of gene targets, respectively (orange = predicted upregulation matched experimental observation, blue = predicted downregulation matched experimental observation, yellow = predicted regulation did not match experimental observation, grey = IPA did not predict target regulation). Red and green shades represent upregulated and downregulated DEGs for that sample group, respectively, when greater intensity of those shades is proportional to greater fold-change. Shapes in figure represent different molecule types that translate/result from the expressed genes (rectangle = ligand-dependent nuclear receptor, square = cytokine, vertical diamond = enzyme, horizontal diamond = peptidase, horizontal oval = transcriptional regulator, vertical oval = transmembrane receptor, isosceles trapezoid = transporter, triangle = kinase, circle = other). **(A)** IPA de novo network for the S1-10 timepoint. PPARa and STAT5b-mediated transcriptional activity are predicted to be repressed (blue) and activated (orange), respectively. **(B)** IPA *de novo* network for the Direct (78-weeks) timepoint. PPARa and STAT5b-mediated transcriptional activity are predicted to be activated (orange) and repressed (blue), respectively.

Supplementary Figure 2 Chromosomal location of DMRs for Stop (S1-78) and Direct groups. For each mouse chromosome, the top figure represents the Stop group, while the bottom represents the Direct group. The *y-axis* represents the difference in methylation compared to control samples (drinking water only) at 78-weeks, where red dots are chromosomal locations of hypermethylated DMRs and blue dots are chromosomal locations of hypomethylated DMRs. Averaged intervals of hyper- or hypomethylation are represented by a gradient scale of red-to-blue, respectively, on the chromosome map (black colored internal represent no DMRs in that interval).
