# Supplementary figures and images for: Persistent gene expression and DNA methylation alterations linked to carcinogenic effects of dichloroacetic acid
Source: Front Oncol. 2024 May 3;14:1389634. doi: 10.3389/fonc.2024.1389634 (PMC11099211; doi:10.3389/fonc.2024.1389634)

Supplemental Figure 1A

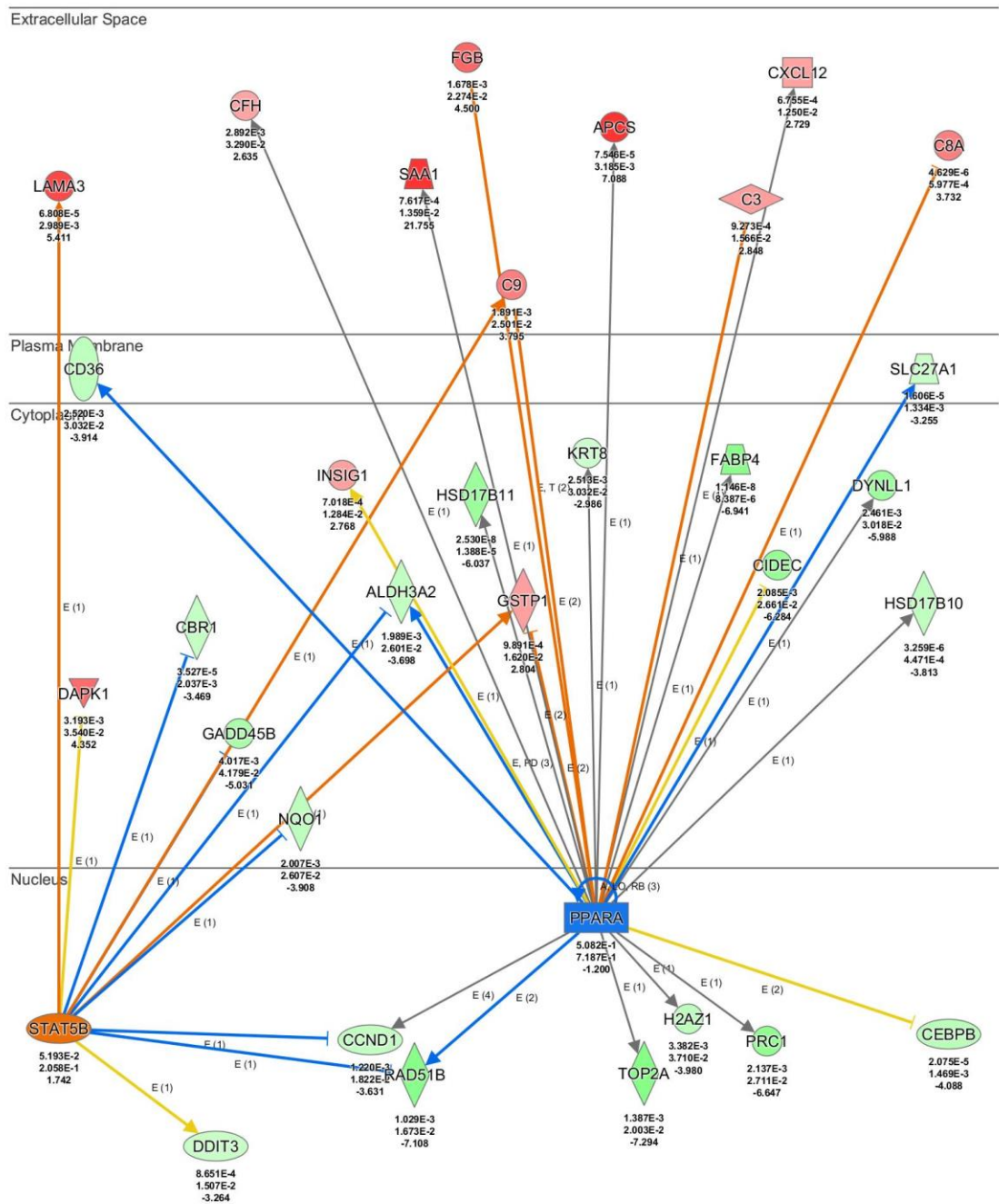

Supplemental Figure 1B

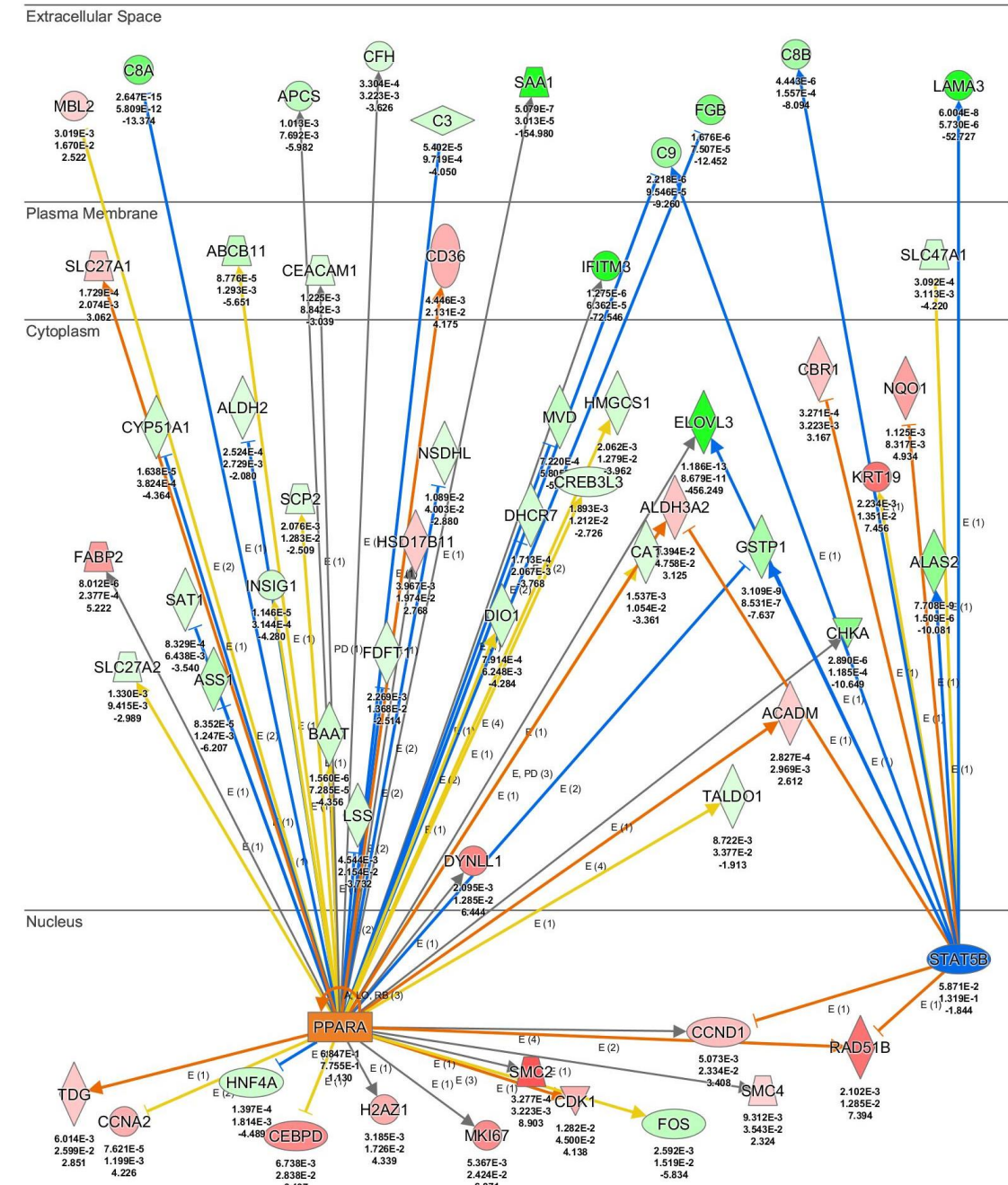

Supplement: Supplementary Figure 1 — Ingenuity Pathway Analysis (IPA) de novo gene network generation based on DEGs from continuous 3.5 g/L DCA exposure in drinking water at 10 and 78 weeks. Arrows and capped lines represent known upregulation and downregulation of gene targets, respectively (orange = predicted upregulation matched experimental observation, blue = predicted downregulation matched experimental observation, yellow = predicted regulation did not match experimental observation, grey = IPA did not predict target regulation). Red and green shades represent upregulated and downregulated DEGs for that sample group, respectively, when greater intensity of those shades is proportional to greater fold-change. Shapes in figure represent different molecule types that translate/result from the expressed genes (rectangle = ligand-dependent nuclear receptor, square = cytokine, vertical diamond = enzyme, horizontal diamond = peptidase, horizontal oval = transcriptional regulator, vertical oval = transmembrane receptor, isosceles trapezoid = transporter, triangle = kinase, circle = other). (A) IPA de novo network for the S1-10 timepoint. PPARa and STAT5b-mediated transcriptional activity are predicted to be repressed (blue) and activated (orange), respectively. (B) IPA de novo network for the Direct (78-weeks) timepoint. PPARa and STAT5b-mediated transcriptional activity are predicted to be activated (orange) and repressed (blue), respectively. [file Image_1.pdf]

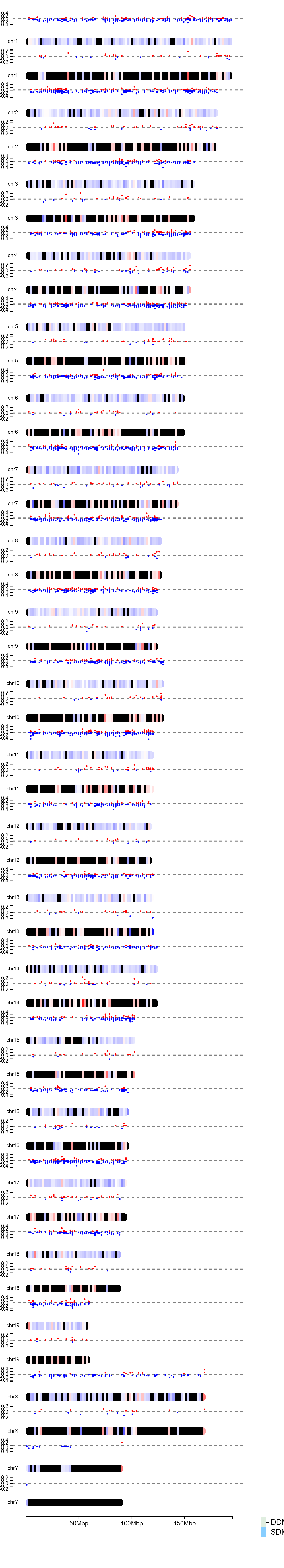

Supplement: Supplementary Figure 2 — Chromosomal location of DMRs for Stop (S1-78) and Direct groups. For each mouse chromosome, the top figure represents the Stop group, while the bottom represents the Direct group. The y-axis represents the difference in methylation compared to control samples (drinking water only) at 78-weeks, where red dots are chromosomal locations of hypermethylated DMRs and blue dots are chromosomal locations of hypomethylated DMRs. Averaged intervals of hyper- or hypomethylation are represented by a gradient scale of red-to-blue, respectively, on the chromosome map (black colored internal represent no DMRs in that interval). [file Image_2.tiff]
